# The Effect of Regeneration on the Adsorptive and Mechanical Properties of WG-12 Granular Activated Carbon Used in Water Treatment: A Case Study

**DOI:** 10.3390/ma19143047

**Published:** 2026-07-15

**Authors:** Katarzyna Ignatowicz, Jacek Piekarski, Łukasz Winconek, Bartosz Dąbrowski

**Affiliations:** 1 Faculty of Civil Engineering and Environmental Sciences, Bialystok University of Technology, Wiejska 45a, 15-351 Białystok, Poland; 2 Faculty of Civil Engineering, Environmental and Geodetic Sciences, Koszalin University of Technology, Śniadeckich 2, 75-453 Koszalin, Poland; jacek.piekarski@tu.koszalin.pl; 3 Grand Activated Sp. z o.o., ul. Białostocka 1, 17-200 Hajnówka, Poland; winconekl@grand-activated.pl; 4 Faculty of Power and Aeronautical Engineering, Warsaw University of Technology, Nowowiejska 24, 00-665 Warsaw, Poland; bartosz.dabrowski8.stud@pw.edu.pl

**Keywords:** WG-12 granular activated carbon, regeneration, water treatment, adsorption

## Abstract

This study evaluated the effectiveness of regenerating WG-12 granular activated carbon used in drinking water treatment, based on a case study of the “Miedwie” Water Production Plant supplying the city of Szczecin, Poland. The investigation was conducted using samples collected from eight adsorption beds after five years of operation. Thermal regeneration was performed in a steam atmosphere at 600–850 °C for 20 min, and the regenerated samples were subsequently demineralized using a 1.75% HCl solution. The effectiveness of the process was evaluated using changes in the iodine number, BET specific surface area, bulk density, mechanical strength, regeneration mass yield, and an adsorption–mechanical trade-off index. Thermal regeneration substantially improved the adsorption properties of the spent carbon. The mean iodine number increased from 528.2 mg/g to 858.1 mg/g after regeneration at 800 °C, while the specific surface area increased from 480.4 to 806.9 m^2^/g. The application of demineralization further enhanced process efficiency. In the 800 °C + 1.75% HCl variant, an iodine number of 903.9 mg/g and a specific surface area of 883.6 m^2^/g were obtained, corresponding to recoveries of 95.1% and 98.2% of the reference values for virgin carbon, respectively. The improvement in adsorption properties was accompanied by a decrease in bulk density and a moderate reduction in mechanical strength. The most favorable process variant was regeneration at 800 °C combined with HCl demineralization, for which the adsorption–mechanical trade-off index reached 93.7%. The results indicate that regeneration of WG-12 effectively restores the adsorption potential of the material and may reduce the need for complete bed replacement while maintaining acceptable mechanical durability. The analysis also indicates that increasing the temperature to 850 °C is not technologically justified because it does not improve adsorption performance and simultaneously increases material loss, which is important from both economic and sustainability perspectives. The proposed I_SM_ index should be regarded as a supplementary screening tool for comparing regeneration variants within the same experimental system, whereas mass yield and cost-related aspects should be evaluated separately to avoid overstating the universality of the index.

## 1. Introduction

Activated carbon is one of the most widely used adsorbent materials in water treatment applications. Its effectiveness is primarily attributed to its well-developed porous structure, high specific surface area, and ability to adsorb a wide range of organic and inorganic contaminants. Granular activated carbon is commonly used for the removal of natural organic matter, micropollutants, pesticides, compounds responsible for taste and odor, and disinfection by-products from water [1,2,3,4,5,6].

During the operation of filtration beds, the adsorption capacity of activated carbon gradually declines. This process is associated with the occupation of active sites by adsorbed compounds, pore blockage, and the accumulation of mineral deposits. As a result, the available adsorption surface area decreases, leading to reduced bed performance. Spent activated carbon may either be replaced with fresh material or subjected to regeneration, which allows partial restoration of its functional properties [7,8,9,10].

Regeneration of spent activated carbon offers significant technological and environmental advantages. It reduces waste generation, lowers the demand for virgin adsorbent, and decreases operating costs associated with bed replacement. Among the available regeneration techniques, thermal regeneration remains the most widely applied because it enables the removal of a substantial fraction of adsorbed substances and promotes reopening of the porous structure. Thermal regeneration was selected in this study because it is one of the most mature and extensively implemented methods for restoring spent granular activated carbon used in water treatment systems. Compared with purely chemical or solvent-based approaches, thermal treatment facilitates the desorption, decomposition, and removal of a broad spectrum of organic compounds accumulated within the pore structure during long-term operation. This is particularly important in drinking water treatment systems, where spent activated carbon contains a complex mixture of natural organic matter, oxidation by-products, taste- and odor-causing compounds, and other adsorbed micropollutants. Nevertheless, the effectiveness of the process depends on the type of activated carbon, the degree of exhaustion, the nature of the adsorbed contaminants, and the applied regeneration conditions [11].

Recent studies have shown that the effectiveness of spent activated carbon regeneration should be interpreted in terms of the combined effects of contaminant desorption, decomposition of adsorbed organic matter, removal of mineral deposits, reopening of micro- and mesopores, and preservation of the carbon framework. Accordingly, recovery of the iodine number and BET specific surface area should be considered together with changes in bulk density, mechanical strength, and surface chemistry rather than being interpreted as isolated engineering indicators [8,12,13,14].

Assessment of regeneration effectiveness should not be limited to improvements in adsorption properties alone. Operational parameters that determine the suitability of activated carbon for reuse in filtration beds are equally important. These include BET specific surface area (S_BET_, m^2^/g), iodine number (IN, mg/g), bulk density (ρ, kg/m^3^), and mechanical strength (MS, %). Only a combined evaluation of these parameters allows determination of whether the regenerated material is suitable for further operation [12,15].

In the literature, regeneration efficiency is commonly evaluated using individual indicators, including adsorption capacity recovery, iodine number recovery, BET surface area recovery, mass yield, ash removal, and mechanical durability. The present study follows this approach by reporting each parameter separately and subsequently applying the I_SM_ index as an auxiliary comparative metric. Consequently, the proposed index is not intended to replace established regeneration criteria but rather to support the selection of a technically balanced regeneration variant when adsorption and mechanical properties are considered simultaneously [11,13,14].

The novelty of this study lies in the integrated evaluation of WG-12 regeneration based on adsorption, physicochemical, and mechanical parameters, combined with the application of an adsorption–mechanical trade-off index for comparative assessment of regeneration variants. This approach enables not only the restoration of adsorption performance to be quantified but also the practical suitability of the regenerated material for reuse in full-scale adsorption beds to be evaluated. The originality of the work is therefore associated primarily with the multi-parameter assessment of spent WG-12 carbon collected from eight full-scale adsorption beds after five years of operation, rather than with the development of a universal regeneration model.

Therefore, the objective of this study was to evaluate the effectiveness of thermal regeneration and subsequent HCl demineralization of spent WG-12 granular activated carbon collected from full-scale drinking water treatment adsorption beds. Particular attention was given to the restoration of adsorption properties, changes in physicochemical and mechanical characteristics, regeneration mass yield, and the identification of operating conditions providing the most favorable balance between adsorption recovery and mechanical durability.

## 2. Materials and Methods

### 2.1. Study Material

The study site was the “Miedwie” Water Production Plant, which forms part of the water supply system of the city of Szczecin, Poland. The plant treats surface water abstracted from Lake Miedwie and constitutes the primary source of drinking water for the city. The treatment train includes pre-ozonation, coagulation, sedimentation, filtration through an anthracite–sand bed, intermediate ozonation, adsorption on granular activated carbon beds, and final disinfection with chlorine dioxide [16]. Owing to the presence of adsorption beds, the facility provides an appropriate case study for evaluating the performance and regeneration of WG-12 granular activated carbon used in water treatment applications.

**Figure 1:**
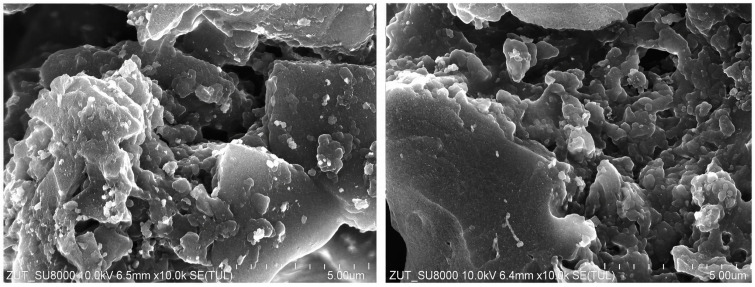
Surface morphology of WG-12 activated carbon observed using SEM [13].

According to the manufacturer’s specifications provided by Grand Activated Sp. z o.o. (Hajnówka, Poland) [17], WG-12 granular activated carbon (Figure 1, Table 1) is an adsorbent designed for water treatment applications. The material is produced from a mixture of hard coal and a binder and is supplied in the form of black cylindrical granules. It is used in water purification and treatment processes, including dechlorination, taste and odor control, and the removal of organic contaminants.

The selection of WG-12 granular activated carbon for this study was based on technological, operational, and practical considerations. First, WG-12 is a commercially available coal-based activated carbon specifically intended for water treatment applications, including the removal of organic contaminants and compounds responsible for undesirable taste and odor. Moreover, WG-12 is widely used in municipal water treatment plants across Poland, which makes it a representative adsorbent for assessing the practical applicability of regeneration strategies in full-scale drinking water treatment systems. Second, the material had been used under real operating conditions in the adsorption beds of the “Miedwie” Water Production Plant, enabling evaluation of regeneration effectiveness for carbon exhausted during long-term, full-scale operation. Third, WG-12 exhibits high reference adsorption characteristics, including an iodine number of at least 950 mg/g and a BET specific surface area of at least 900 m^2^/g, making it suitable for assessing the degree of adsorption-property recovery after regeneration. Finally, the use of the same carbon type in multiple adsorption beds enabled comparison of regeneration responses among samples exposed to similar treatment conditions but originating from different operational units.

SEM images of WG-12 activated carbon (Figure 1) reveal a surface morphology characteristic of hard-coal-derived activated carbons. The material exhibits a highly developed, irregular, and heterogeneous surface containing numerous fractures, fissures, microcracks, and angular plate-like structures. Local depressions, surface irregularities, and fine particles deposited on larger grain fragments are also visible. Such morphology indicates a complex surface structure that is favorable for adsorption processes.

The study was conducted using samples of WG-12 granular activated carbon collected after five years of service from eight adsorption beds located in Szczecin, Poland, operating at the “Miedwie” Water Production Plant. Sampling and sample preparation were performed in accordance with the general principles of representative sampling of granular materials specified in ISO 1988:1975 [18].

The eight samples collected from adsorption beds A–H were treated as independent operational units representing variability among full-scale beds. They should not be interpreted as analytical replicates of a single homogenized laboratory sample. Accordingly, the reported mean values describe the average regeneration response of activated carbon from eight operating beds, whereas the observed ranges reflect bed-to-bed variability resulting from long-term operation under real drinking-water treatment conditions. For every regeneration temperature and treatment variant, three separate experimental runs were performed under identical conditions, and the reported values correspond to the arithmetic mean of these independent experimental runs.

### 2.2. Preparation of Samples for Analysis

Before laboratory analyses were performed, the spent activated carbon samples were dried and sieved to remove dust and fine fractions. The purpose of this procedure was to homogenize the material and minimize the influence of moisture and fine particles on the results of physicochemical analyses. The prepared material served as the initial sample for evaluating carbon properties before regeneration, after regeneration, and after the subsequent demineralization process.

### 2.3. Laboratory Regeneration of Activated Carbon

**Figure 2:**
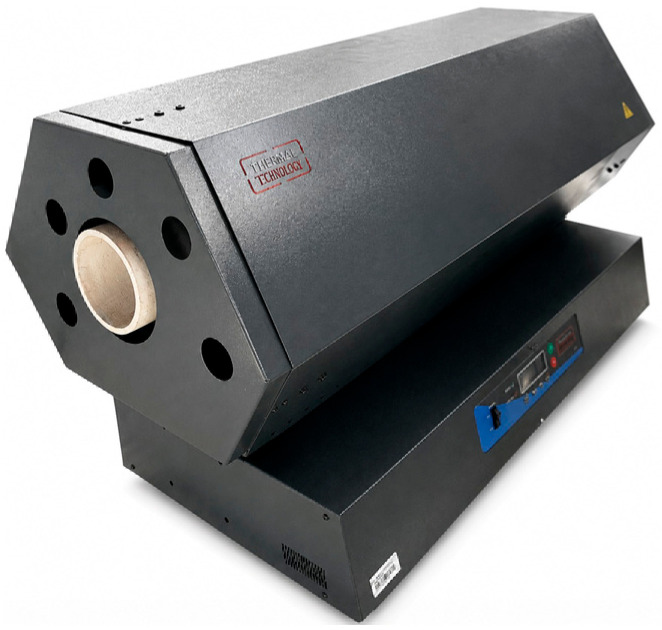
MTTF-1200 tube furnace manufactured by MagmaTherm.

Laboratory regeneration of WG-12 granular activated carbon was carried out under controlled conditions using a laboratory-scale system designed for thermal regeneration of carbonaceous adsorbents. The experimental setup consisted of an MTTF-1200 tube furnace manufactured by MagmaTherm (Istanbul, Türkiye) (Figure 2), a BINDER ED-56 (BINDER GmbH, Tuttlingen, Germany) laboratory dryer with natural air circulation, a MultiSerw-Morek (Brzeźnica, Poland) vacuum sieve shaker, a steam dosing system comprising a pump and a heater, a Radwag (Radom, Poland) laboratory balance, and a computer used for process monitoring, recording, and visualization.

The regeneration process was performed using a thermal method in a steam atmosphere. Steam acted both as a protective medium and as a reactive agent, promoting degassing of the material, desorption of contaminants accumulated during operation, and partial reopening of the porous structure of the activated carbon. Regeneration was conducted in a tube furnace, where activated carbon samples were placed in the heating zone and exposed to the target temperature under a continuous steam flow.

Before each experiment, the furnace was stabilized at the selected operating temperature. Steam was introduced during the isothermal regeneration stage, and identical steam-dosing settings were maintained for all temperature variants. The regeneration time of 20 min corresponded to the duration of isothermal exposure of the carbon sample under a steam atmosphere. The steam environment limited direct oxidation of the carbon matrix while promoting desorption, degassing, and partial gasification of residues blocking the porous structure.

For each experiment, a constant feed mass of m_0_ = 200 g and a constant regeneration time of 20 min were adopted. The regeneration temperature, T [°C], was the only variable process parameter. Experiments were performed at six temperature levels: 600, 650, 700, 750, 800, and 850 °C. This temperature range enabled evaluation of the effect of thermal treatment intensity on the restoration of adsorption properties and on changes in operational parameters, including bulk density and mechanical strength. Temperature was intentionally selected as the primary experimental variable, whereas steam dose, sample mass, and residence time were kept constant to isolate the influence of temperature on adsorption recovery and material degradation.

After regeneration, the samples were cooled and subsequently sieved to remove dust and fine fractions generated during the process. The resulting material constituted the thermally regenerated carbon sample. Basic physicochemical parameters were then determined for each regeneration variant.

### 2.4. Demineralization of Regenerated Samples

Following thermal regeneration, the samples were subjected to demineralization using a 1.75% HCl solution. The contact time was 2 h, the sample-to-solution ratio was maintained at 1:10, and the process temperature was kept within the range of 20–25 °C. The objective of this stage was to reduce the content of mineral matter and ash deposits present within the pores and on the surface of the activated carbon granules. After acid treatment, the samples were rinsed with distilled water until a stable filtrate pH was achieved and were subsequently dried to constant mass.

The concentration of 1.75% HCl was selected as a relatively mild demineralization condition. The purpose of this treatment was to dissolve and remove mineral and ash deposits accumulated within the pore system while minimizing excessive chemical attack on the carbon matrix. Since acid concentration was not investigated as an independent experimental variable, the results should be interpreted as an evaluation of a single post-treatment option rather than a comprehensive optimization of acid-washing conditions.

The properties of carbon subjected to thermal regeneration alone and to regeneration followed by demineralization were compared with those of spent carbon and with the reference values for virgin WG-12 activated carbon. This approach enabled assessment of the degree of restoration of operational properties and identification of the most effective regeneration variant.

### 2.5. Scope of Physicochemical Determinations

Regeneration effectiveness was evaluated based on changes in selected physicochemical and mechanical properties of the activated carbon. All analyses of virgin, spent, and regenerated WG-12 samples were conducted in the accredited analytical laboratory of Grand Activated Sp. z o.o. (Poland), the manufacturer of the adsorbent. The analytical program was designed to provide a comprehensive assessment of regeneration performance, taking into account both the recovery of adsorption properties and the preservation of the material’s mechanical integrity.

The following parameters were determined:

- BET specific surface area (S_BET_, m^2^/g), according to PN-ISO 9277:2000 [19];
- Iodine number (IN, mg/g), according to PN-EN 12902:2005 [20];
- Bulk density (ρ, kg/m^3^), according to PN-EN 12915-1:2009 [21];
- Mechanical strength (MS, %), according to PN-EN 12915-1:2009 [21].

These parameters characterize both the restored adsorption potential and the operational suitability of regenerated activated carbon. Direct adsorption experiments involving natural organic matter, disinfection by-product precursors, pesticides, or other micropollutants were beyond the scope of the present study. Consequently, IN and S_BET_ should be interpreted as indirect indicators of adsorption potential rather than direct measures of contaminant-removal efficiency in treated water.

For each investigated temperature, all analyzed response parameters were determined in triplicate for each of the eight adsorption beds. Therefore, the reported values account for both the variability among full-scale adsorption beds and the repeatability of laboratory determinations.

### 2.6. Mass Yield of Regeneration

Mass-yield analysis was performed for samples collected from adsorption beds A–H at the “Miedwie” Water Production Plant and regenerated at temperatures ranging from 600 to 850 °C. For each temperature variant, the initial sample mass was maintained at m_0_ = 200 g, whereas the mass after regeneration was calculated as the mean value obtained for beds A–H.

Mass yield was included as a separate indicator of material efficiency because it directly determines the quantity of regenerated carbon that can be returned to the adsorption bed. This parameter is also important from an economic perspective, as high adsorption recovery may be less attractive in practice if accompanied by excessive material loss.

(1) $$\eta_{R}=\left(m_{R}/m_{0}\right)\cdot 100\%,$$

where

η_R_—mass field of regeneration, [%];

m_R_—mass after regeneration, [g];

m_0_—initial sample mass, [g].

### 2.7. Adsorption–Mechanical Trade-Off Index Calculation

To provide a synthetic assessment of WG-12 regeneration effectiveness, an adsorption–mechanical trade-off index (I_SM_, %) was proposed. This index simultaneously considers restoration of adsorption properties, expressed by the iodine number (IN) and BET specific surface area (S_BET_), together with retention of mechanical strength (MS) following regeneration.

The adsorption–mechanical trade-off index was defined as follows:

(2) $$I_{\mathrm{SM}}=\;100\cdot R_{S}\cdot R_{M},$$

where

(3) $$R_{S}=\frac{1}{2}\left(\frac{{\mathrm{IN}}_{R}}{{\mathrm{IN}}_{N}}+\frac{S_{\mathrm{BET},R}}{S_{\mathrm{BET},N}}\right)\;{\mathrm{and}\;R}_{M}=\frac{MS_{R}}{MS_{0}},$$

where

I_SM_—adsorption-mechanical trade-off index, [%];

R_S_—relative degree of restoration of adsorption properties, [-];

R_M_—relative retention of mechanical strength, [-];

IN_R_—iodine number after regeneration or after regeneration combined with demineralization, [mg/g];

IN_N_—iodine number of virgin carbon, [mg/g];

S_BET,R_—specific surface area after regeneration or after regeneration combined with demineralization, [m^2^/g];

S_BET,N_—specific surface area of virgin carbon, [m^2^/g];

MS_R_—mechanical strength after regeneration or after regeneration combined with demineralization, [%];

MS_0_—mechanical strength of spent carbon before regeneration, [%].

The use of this index is justified because evaluation of regeneration effectiveness should not rely exclusively on improvements in adsorption properties. From a practical standpoint, the most desirable regeneration variant is one that achieves high IN and S_BET_ values while maintaining sufficient mechanical integrity of the granules. Excessively severe regeneration conditions may enhance adsorption performance but simultaneously weaken the grain structure, thereby limiting the suitability of the material for reuse in adsorption beds.

The multiplicative form of the I_SM_ index was selected because it penalizes unbalanced regeneration outcomes. A material exhibiting high adsorption recovery but poor mechanical durability would have limited practical value, while a mechanically stable material with inadequate adsorption performance would not provide satisfactory treatment efficiency. Consequently, the multiplicative formulation reflects the requirement that both adsorption and mechanical characteristics should remain at high levels simultaneously.

No weighting factors were introduced because the study did not assume a priori preference for adsorption recovery over mechanical durability, or vice versa. Equal weighting was adopted to avoid arbitrary prioritization of individual performance criteria. Nevertheless, in full-scale applications, weighted indices or multi-criteria decision-making approaches may be more appropriate when operational priorities are clearly defined.

It should also be emphasized that the I_SM_ index does not include mass yield or treatment costs. Therefore, I_SM_ is used in this study solely as an adsorption–mechanical screening metric, whereas regeneration mass yield is evaluated separately as an indicator of material efficiency and economic relevance. A more comprehensive techno-economic index could additionally incorporate adsorption recovery, mechanical durability, mass yield, energy consumption, chemical consumption, and expected regeneration-cycle life. However, these factors were beyond the scope of the present investigation.

### 2.8. Surface Chemistry and Morphology Characterization

Fourier-transform infrared spectroscopy (FTIR) was used to characterize the chemical properties of the activated carbon surface. Measurements were carried out with an Alpha FTIR spectrometer (Bruker Optics GmbH & Co. KG, Ettlingen, Germany). The spectra were collected in the range of 4000–400 cm^−1^, with a spectral resolution of 1 cm^−1^. For each spectrum, 64 scans were recorded and averaged to improve the signal quality. Before analysis, the activated carbon samples were finely ground in an agate mortar and homogenized. The FTIR specimens were prepared by mixing 1 mg of powdered activated carbon with 200 mg of dried potassium bromide (KBr). The mixture was then pressed into pellets using a Specac hydraulic press (Specac Ltd., Orpington, UK). A pellet made of pure KBr was applied as the background reference.

Scanning electron microscopy (SEM) was applied to examine the morphology of the activated carbon granules and to identify changes caused by operation and regeneration. Particular attention was paid to surface deposits, contamination, pore accessibility, structural irregularities, and changes in the external texture of the granules. SEM observations were performed using a Quanta FEG 250 scanning electron microscope (FEI Company, Hillsboro, OR, USA). Prior to imaging, carbon granules were fixed onto aluminum stubs with conductive carbon adhesive tape, which ensured electrical contact and reduced charge accumulation on the sample surface. The observations were conducted under high-vacuum conditions at an accelerating voltage of 10 kV. Images were recorded using a secondary electron detector. For each sample, micrographs were taken at different magnifications, from 50× to 40,000×. Representative images were selected for further assessment of surface topography, microstructural characteristics, and visible porosity.

## 3. Results and Discussion

**Figure 3:**
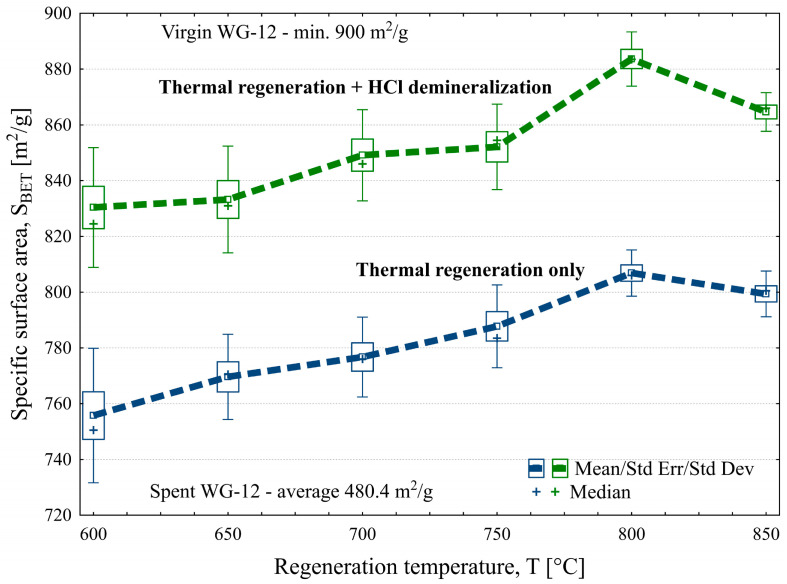
Changes in the BET specific surface area, S_BET_ [m^2^/g], of WG-12 granular activated carbon from adsorption beds A–H at the “Miedwie” Water Production Plant as a function of regeneration temperature, T [°C], and demineralization with a 1.75% HCl solution.

**Figure 4:**
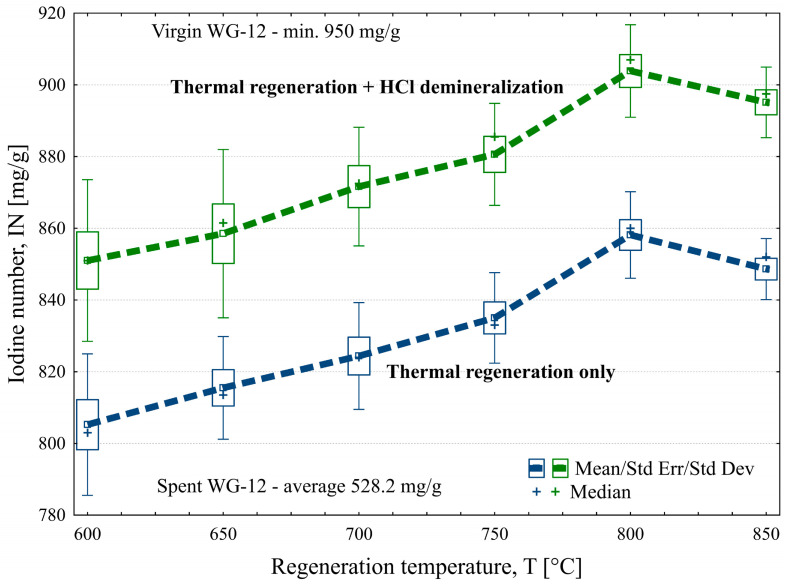
Changes in the iodine number, IN [mg/g], of WG-12 granular activated carbon from adsorption beds A–H at the “Miedwie” Water Production Plant as a function of regeneration temperature, T [°C], and demineralization with a 1.75% HCl solution.

**Figure 5:**
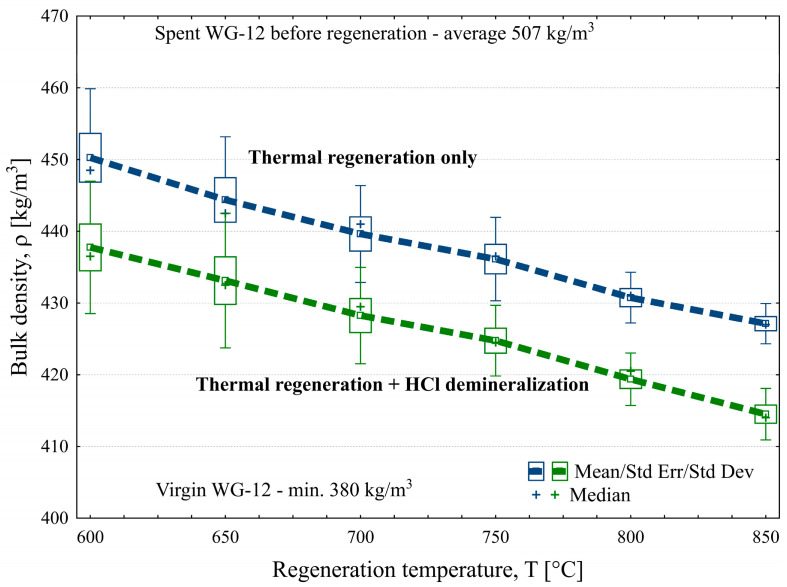
Changes in the bulk density, ρ [kg/m^3^], of WG-12 granular activated carbon from adsorption beds A–H at the “Miedwie” Water Production Plant as a function of regeneration temperature, T [°C], and demineralization with a 1.75% HCl solution.

**Figure 6:**
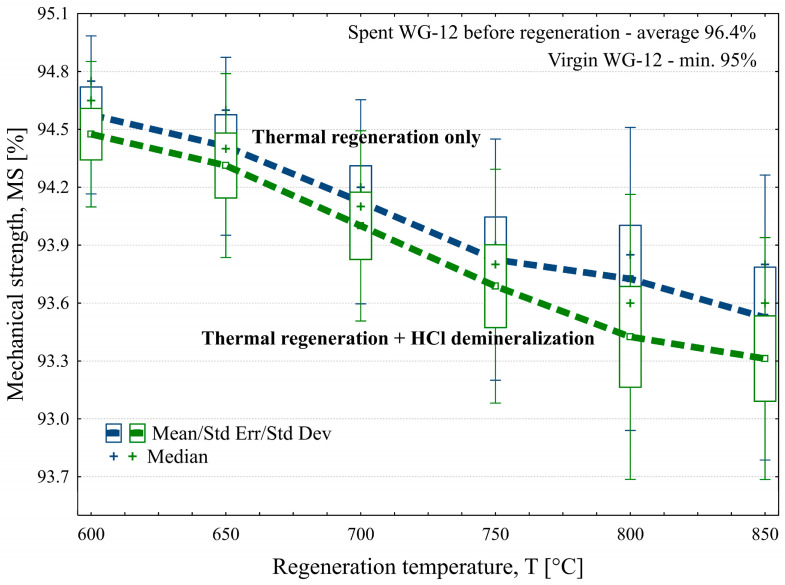
Changes in the mechanical strength, MS [%], of WG-12 granular activated carbon from adsorption beds A–H at the “Miedwie” Water Production Plant as a function of regeneration temperature, T [°C], and demineralization with a 1.75% HCl solution.

**Figure 7:**
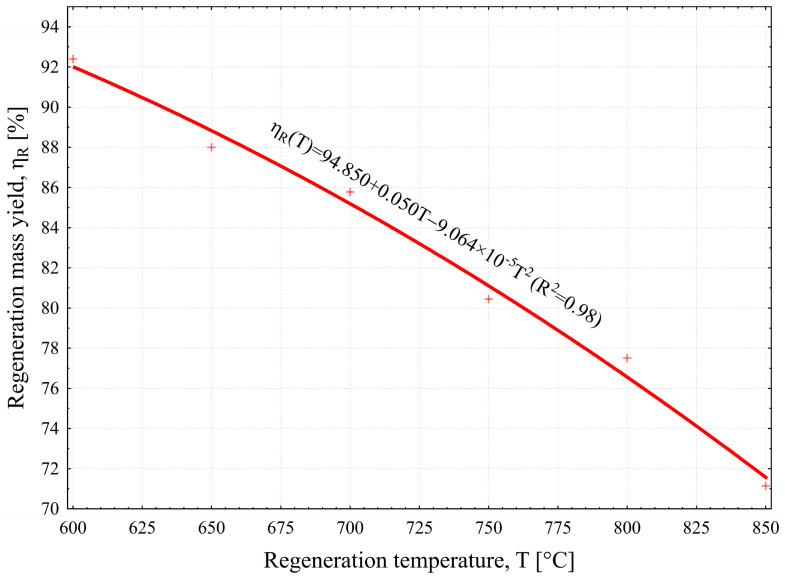
Effect of regeneration temperature, T [°C], on the regeneration mass yield, η_R_ [%], of WG-12 granular activated carbon from adsorption beds at the “Miedwie” Water Production Plant.

Based on the experimental results, a series of plots was prepared to illustrate the influence of regeneration temperature, T [°C], on the analyzed response variables. The corresponding relationships are presented in Figure 3, Figure 4, Figure 5, Figure 6 and Figure 7.

Unless stated otherwise, all values discussed in this section represent mean values calculated from samples collected from eight adsorption beds. The observed variability reflects operational differences among full-scale adsorption beds rather than analytical repeatability. This distinction is important because the study was designed as a case study based on activated carbon exhausted under real operating conditions rather than as a controlled laboratory repeatability experiment.

### 3.1. BET Specific Surface Area, S_BET_

Analysis of the results demonstrated a clear influence of regeneration temperature on the BET specific surface area (S_BET_) of WG-12 granular activated carbon collected from adsorption beds A–H at the “Miedwie” Water Production Plant. For all analyzed samples, thermal regeneration resulted in a substantial increase in S_BET_ compared with the spent carbon (Figure 3).

The mean S_BET_ value of the spent carbon prior to regeneration was 480.4 m^2^/g. Following thermal regeneration, this value increased to 755.8 m^2^/g at 600 °C and reached a maximum of 806.9 m^2^/g at 800 °C. This corresponds to an increase of 326.5 m^2^/g, or approximately 68%, relative to the spent carbon. At 850 °C, the mean S_BET_ value decreased slightly to 799.4 m^2^/g, although it remained substantially higher than that of the untreated material. The increase in S_BET_ can be attributed to the removal of substances adsorbed during bed operation, thermal degassing, and partial reopening of the porous structure [9,10,17,22]. This interpretation is supported by the simultaneous increase in the iodine number (IN), indicating improved adsorption performance of the regenerated activated carbon.

Additional demineralization using a 1.75% HCl solution further enhanced S_BET_ across all temperature variants. After demineralization, mean S_BET_ values ranged from 830.4 m^2^/g at 600 °C to 883.6 m^2^/g at 800 °C. Compared with thermal regeneration alone, acid treatment increased S_BET_ by approximately 63.6–76.8 m^2^/g, depending on regeneration temperature.

The most favorable results were obtained at 800 °C, both for thermal regeneration alone and for regeneration followed by demineralization. Increasing the temperature to 850 °C did not provide any further improvement. Instead, S_BET_ decreased from 806.9 to 799.4 m^2^/g after thermal regeneration and from 883.6 to 864.6 m^2^/g after demineralization. These findings suggest that regeneration temperatures above 800 °C do not contribute to additional development of accessible adsorption surface area.

The increase in S_BET_ was accompanied by a decrease in bulk density (ρ), which may be attributed to the removal of contaminants and mineral deposits from pores and grain surfaces, resulting in a greater proportion of accessible pore volume. In this context, a moderate reduction in ρ should not necessarily be interpreted as deterioration of material quality but rather as evidence of pore cleaning and increased accessibility of adsorption sites. Nevertheless, bulk density should be evaluated together with mechanical strength (MS), as excessive reductions in ρ may indicate structural weakening of the granules [11,12,13,23].

### 3.2. Iodine Number, IN

Analysis of changes in the iodine number (IN) also demonstrated a pronounced effect of regeneration temperature on restoration of the adsorption properties of WG-12 activated carbon. For all samples collected from adsorption beds A–H, thermal regeneration resulted in higher IN values compared with those measured for the spent carbon (Figure 4).

The mean IN value before regeneration was 528.2 mg/g. Following regeneration at 600 °C, it increased to 805.2 mg/g, while the highest value obtained after thermal regeneration alone was recorded at 800 °C, reaching 858.1 mg/g. This represents an increase of 329.9 mg/g, corresponding to approximately 62.5% relative to the spent material. A further increase in temperature to 850 °C did not improve regeneration performance, as the mean IN value decreased slightly to 848.6 mg/g.

The increase in IN can be attributed to removal of adsorbed substances, reopening of microporous structures, and increased accessibility of adsorption sites within the carbon matrix [14]. Because the iodine number is primarily associated with microporosity, its increase provides strong evidence for restoration of the porous structure. This interpretation is consistent with the manufacturer’s specification indicating an iodine number of at least 950 mg/g for virgin WG-12 activated carbon.

Demineralization with a 1.75% HCl solution resulted in an additional increase in IN for all temperature variants. After acid treatment, mean values ranged from 851.0 mg/g at 600 °C to 903.9 mg/g at 800 °C. Consequently, the most favorable performance was again observed at 800 °C, where regeneration followed by demineralization increased IN by 375.7 mg/g, corresponding to approximately 71.1% relative to the spent carbon.

The results indicate that 800 °C was the optimal temperature for restoring the adsorption capacity of WG-12 activated carbon under the investigated conditions. At this temperature, the highest IN values were obtained both before and after demineralization. Increasing the regeneration temperature to 850 °C failed to provide additional improvement and was accompanied by a further reduction in bulk density (ρ), suggesting that higher process intensity was not technologically justified.

### 3.3. Bulk Density, ρ

The results demonstrated a clear influence of regeneration temperature on the bulk density (ρ) of WG-12 granular activated carbon collected from adsorption beds A–H at the “Miedwie” Water Production Plant. A systematic decrease in bulk density was observed throughout the investigated temperature range of 600–850 °C (Figure 5).

The mean bulk density of the spent carbon prior to regeneration was 507.0 kg/m^3^. Following thermal regeneration, this value decreased to 450.3 kg/m^3^ at 600 °C and to 427.1 kg/m^3^ at 850 °C. This corresponds to a reduction of 23.2 kg/m^3^ between the lowest and highest regeneration temperatures, representing approximately 5.1% of the value measured at 600 °C. Among the analyzed adsorption beds, the largest decrease in bulk density was observed for bed F, where ρ declined from 467 to 427 kg/m^3^, whereas the smallest reduction occurred in bed B, where the decrease amounted to 12 kg/m^3^.

The observed decrease in ρ with increasing regeneration temperature can be attributed to intensified thermal processes occurring within the activated carbon structure. Elevated temperatures promote more effective removal of adsorbed contaminants, thermal degassing, and reopening of the pore network. These processes result in mass loss and an increase in the proportion of accessible pores, which in turn reduces bulk density. However, changes in ρ should be interpreted together with mechanical strength (MS), since excessive density reduction may also indicate partial degradation of the granule structure [12,24,25].

As regeneration temperature increased, variability among individual adsorption beds decreased. At 600 °C, bulk-density values ranged from 436 to 467 kg/m^3^, whereas at 850 °C the range narrowed to 423–431 kg/m^3^. This observation suggests that higher regeneration temperatures produced a more uniform regeneration effect, regardless of differences in the initial condition of the samples.

Demineralization with a 1.75% HCl solution resulted in an additional decrease in bulk density. After acid treatment, mean ρ values ranged from 437.8 kg/m^3^ at 600 °C to 414.5 kg/m^3^ at 850 °C. Compared with thermal regeneration alone, this represents an additional reduction of approximately 11–13 kg/m^3^, corresponding to about 2.5–3.0%.

The decrease in ρ after demineralization is most likely associated with dissolution and removal of mineral matter and ash deposits located within the pore structure and on granule surfaces. Acid washing facilitates leaching of inorganic constituents, resulting in additional mass loss and further cleaning of the porous structure. Consequently, lower bulk density values were accompanied by increased accessibility of adsorption sites, reflected in higher S_BET_ values. Nevertheless, interpretation of this parameter should always consider concurrent changes in mechanical strength (MS) [26,27].

### 3.4. Mechanical Strength, MS

The results indicate that thermal regeneration influenced the mechanical strength (MS) of WG-12 granular activated carbon; however, the magnitude of these changes was considerably smaller than that observed for adsorption-related parameters such as IN and S_BET_ (Figure 6).

The mean mechanical strength of the spent carbon prior to regeneration was 96.4%. Following thermal regeneration, MS decreased to 94.6% at 600 °C and to 93.5% at 850 °C. Thus, increasing regeneration temperature resulted in a gradual but relatively modest reduction in mechanical durability. The difference between the lowest and highest values was only 1.1 percentage points.

The largest decrease in mechanical strength was observed for bed B, where MS declined from 94.1% to 91.7% over the investigated temperature range. In contrast, bed A exhibited the smallest change, with mechanical strength decreasing only from 94.1% to 93.8%. Despite these reductions, most regenerated samples maintained MS values above 93%, indicating limited structural degradation of the granules.

The reduction in mechanical strength observed at elevated temperatures is likely related to the effects of thermal treatment on the internal structure of activated carbon particles. During regeneration, degassing, contaminant removal, and pore cleaning promote restoration of adsorption properties. At the same time, excessive thermal exposure may weaken the carbon framework, increase brittleness, and enhance susceptibility to abrasion. These findings highlight the importance of simultaneously evaluating adsorption performance and mechanical durability when assessing regeneration effectiveness [2,12,24].

Additional demineralization with a 1.75% HCl solution caused only a slight further decrease in MS. Mean mechanical strength values after acid treatment ranged from 94.5% at 600 °C to 93.3% at 850 °C. Relative to thermal regeneration alone, the differences were small, averaging only 0.1–0.3 percentage points. This indicates that the demineralization step did not significantly contribute to additional mechanical degradation of the activated carbon.

Mechanical-strength results should be interpreted together with changes in IN, S_BET_, and ρ. The most favorable adsorption performance was achieved at 800 °C, where mean mechanical strength remained at 93.7% after thermal regeneration and 93.4% after regeneration followed by demineralization. Although these values were slightly lower than those of the spent material, they indicate that the regenerated carbon retained satisfactory resistance to abrasion and fragmentation.

### 3.5. Mass Yield of Regeneration, η_R_

Mass-balance analysis demonstrated that increasing regeneration temperature resulted in a systematic reduction in the amount of recovered activated carbon (Figure 7). Based on Equation (1), the regeneration mass yield (η_R_) decreased from 92.40% at 600 °C, corresponding to a mean recovered mass of 184.80 g, to 71.14% at 850 °C, where the mean recovered mass was 142.29 g. As temperature increased, a gradual decline in the amount of recovered material was observed.

The relationship between regeneration temperature and mass yield was accurately described by a second-order polynomial model with a coefficient of determination of R^2^ = 0.98. The high value of R^2^ indicates excellent agreement between the fitted model and the experimental data.

The decrease in mass yield with increasing temperature can be attributed to intensified removal of adsorbed organic compounds and volatile constituents, as well as partial burn-off of the carbon matrix. While these processes contribute to restoration of adsorption properties, they simultaneously reduce the quantity of material available for reuse.

From a practical perspective, these results emphasize the importance of selecting an appropriate regeneration temperature. Insufficient temperatures may fail to provide adequate recovery of adsorption performance, whereas excessively high temperatures lead to substantial material losses, lower recovery yields, and reduced economic attractiveness of the regeneration process.

Taken together with the adsorption and mechanical-property results, the mass-yield analysis indicates that regeneration temperatures above 800 °C provide limited additional benefits while significantly increasing material losses. Consequently, 800 °C appears to represent the most balanced operating condition among the investigated regeneration variants.

### 3.6. Analysis of Selected Correlations

**Figure 8:**
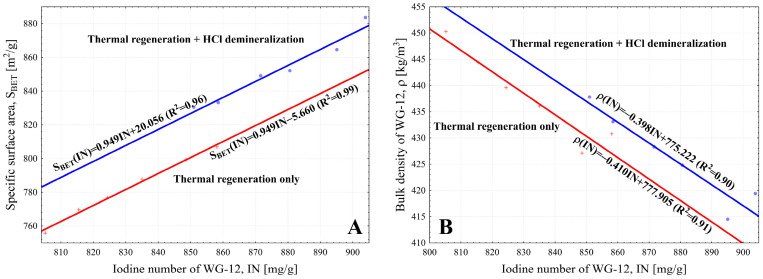
Relationship between iodine number, IN [mg/g], and BET specific surface area, S_BET_ [m^2^/g] (**A**), and between iodine number, IN [mg/g], and bulk density, ρ [kg/m^3^] (**B**), of WG-12 granular activated carbon after thermal regeneration and after thermal regeneration combined with demineralization using a 1.75% HCl solution.

Analysis of the relationship between the iodine number (IN) and BET specific surface area (S_BET_) revealed a very strong positive correlation for both thermally regenerated samples and samples subjected to regeneration followed by demineralization with a 1.75% HCl solution (Figure 8A).

For thermally regenerated carbon, the increase in IN from 805.2 mg/g at 600 °C to 858.1 mg/g at 800 °C was accompanied by an increase in S_BET_ from 755.8 to 806.9 m^2^/g. At 850 °C, both parameters decreased slightly, reaching 848.6 mg/g and 799.4 m^2^/g, respectively. A similar trend was observed after demineralization. In this case, IN increased from 851.0 mg/g at 600 °C to 903.9 mg/g at 800 °C, while S_BET_ increased from 830.4 to 883.6 m^2^/g. Further increasing the temperature to 850 °C resulted in slight decreases in both parameters, with IN and S_BET_ reaching 895.1 mg/g and 864.6 m^2^/g, respectively.

These results demonstrate that the highest adsorption-related parameters were achieved at 800 °C, regardless of whether demineralization was applied.

The observed relationship can be explained by the mechanism of thermal regeneration. Increasing the temperature up to 800 °C promoted removal of adsorbed contaminants, decomposition products, and pore-blocking deposits accumulated during long-term operation. As a result, previously inaccessible micro- and mesopores became available for adsorption, leading to simultaneous increases in both IN and S_BET_. The additional demineralization step further enhanced this effect through removal of mineral and ash deposits from pore walls and grain surfaces. At 800 °C, demineralization increased IN by 45.8 mg/g and S_BET_ by 76.7 m^2^/g compared with thermal regeneration alone.

The relationship between the iodine number (IN) and bulk density (ρ) exhibited a clear negative correlation for both regeneration pathways (Figure 8B). During thermal regeneration, IN increased from 805.2 mg/g at 600 °C to 858.1 mg/g at 800 °C, whereas bulk density decreased from 450.3 to 430.8 kg/m^3^. A similar pattern was observed after demineralization, where IN increased from 851.0 to 903.9 mg/g, while ρ decreased from 437.8 to 419.4 kg/m^3^.

This inverse relationship complements the positive correlation observed between IN and S_BET_. Together, these findings indicate that restoration of adsorption properties was associated with progressive cleaning and reopening of the pore structure. Removal of adsorbed compounds and mineral deposits increased accessibility of the internal surface area, resulting in higher IN and S_BET_ values while simultaneously reducing bulk density.

Demineralization amplified this effect by eliminating additional inorganic deposits from both the external surface and internal pore structure of the activated carbon. Consequently, demineralized samples consistently exhibited higher adsorption parameters and lower bulk-density values than samples subjected solely to thermal regeneration.

At 850 °C, bulk density continued to decrease, whereas no further improvement in adsorption parameters was observed. This finding suggests that excessive process intensity primarily promoted material loss and structural modification rather than additional enhancement of adsorption performance. Combined with the results presented in Figure 8A, these observations indicate that the most favorable regeneration conditions were achieved at approximately 800 °C.

Overall, the correlation analysis supports the proposed regeneration mechanism. Increasing the regeneration temperature up to 800 °C improved accessibility of the pore system and restored adsorption performance, whereas further temperature increases mainly intensified material loss and structural degradation without providing proportional adsorption benefits. These findings are consistent with previous reports indicating that excessive thermal treatment may promote carbon burn-off and weakening of the carbon framework rather than additional recovery of adsorption capacity [11,12,13,14,24].

### 3.7. Assessment of Regeneration Effectiveness

The adsorption–mechanical trade-off index (I_SM_) was calculated using Equations (2) and (3). Reference values for virgin WG-12 activated carbon were adopted as IN = 950 mg/g and S_BET_ = 900 m^2^/g. The mean mechanical strength of the spent carbon before regeneration was MS = 96.4%.

**Figure 9:**
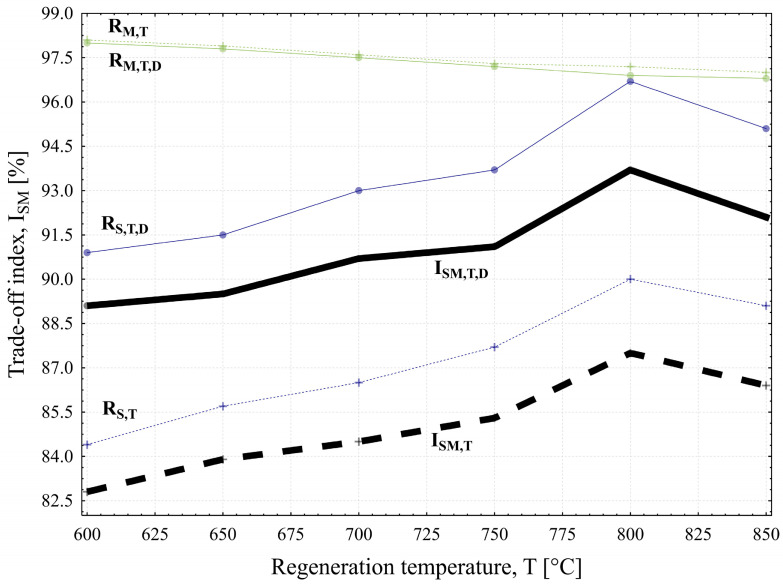
Changes in the trade-off index, I_SM_ [%], of WG-12 granular activated carbon from adsorption beds A–H at the “Miedwie” Water Production Plant as a function of regeneration temperature, T [°C], and demineralization with a 1.75% HCl solution.

Analysis of the results demonstrated that increasing regeneration temperature improved the balance between adsorption-property recovery and mechanical-strength retention up to an optimum temperature of 800 °C (Figure 9).

For thermal regeneration alone, the I_SM,T_ value increased from 82.8% at 600 °C to 87.5% at 800 °C. Further increasing the temperature to 850 °C reduced the index to 86.4%, indicating that additional process intensification did not improve overall regeneration performance.

The increase in I_SM,T_ was primarily driven by improvements in IN and S_BET_, whereas mechanical strength decreased only slightly and therefore had a relatively limited impact on the final index value. Consequently, regeneration at 800 °C provided the most favorable balance between adsorption recovery and mechanical durability among the investigated thermal-regeneration variants.

Application of demineralization using a 1.75% HCl solution further improved regeneration effectiveness. Across the entire temperature range, I_SM,T,D_ values were consistently higher than those obtained after thermal regeneration alone. This improvement resulted from additional increases in IN and S_BET_ following removal of mineral deposits, while mechanical-strength losses remained minimal.

The highest index value was obtained for regeneration at 800 °C followed by demineralization. Under these conditions, the mean degree of adsorption-property restoration reached 96.7%, mechanical-strength retention reached 96.9%, and the resulting I_SM,T,D_ value was 93.7%.

These results indicate that this regeneration pathway provided the most advantageous combination of adsorption recovery and mechanical durability. In practical terms, it represents the most balanced compromise between restoration of adsorption performance and preservation of the structural integrity required for continued operation in full-scale adsorption beds.

At 850 °C, despite relatively high IN and S_BET_ values, the I_SM,T,D_ value decreased to 92.1%. This reduction resulted from slight deterioration of adsorption parameters compared with those obtained at 800 °C, together with additional losses in mechanical strength. Therefore, increasing regeneration temperature beyond 800 °C did not provide any meaningful technological advantage.

The ranking obtained using the I_SM_ index was consistent with conclusions derived from the individual evaluation criteria commonly applied in activated-carbon regeneration studies. Maximum values of IN and S_BET_, acceptable mechanical-strength retention, and the absence of further adsorption improvement at 850 °C all identified the same optimal process region, namely approximately 800 °C. Accordingly, the proposed I_SM_ index should be regarded as a complementary decision-support tool rather than a replacement for conventional performance indicators. When selecting a regeneration strategy for practical applications, I_SM_ should be interpreted together with mass yield, economic considerations, and operational requirements.

### 3.8. Preliminary Cost and Sustainability Considerations in Regeneration Temperature Selection

Although a detailed techno-economic analysis was beyond the scope of this study, the obtained results enable a preliminary assessment of the economic and sustainability implications associated with regeneration temperature selection. In practical applications, the feasibility of activated carbon regeneration depends not only on the recovery of adsorption properties but also on process yield, energy consumption, chemical usage, and the quantity of material that can be returned to service. Consequently, the optimal regeneration temperature should represent a balance between adsorption performance, mechanical integrity, material recovery, and process intensity.

The results demonstrated that increasing the regeneration temperature from 600 to 800 °C significantly improved the adsorption properties of WG-12 activated carbon. The iodine number increased from 805.2 to 858.1 mg/g after thermal regeneration and from 851.0 to 903.9 mg/g after regeneration combined with HCl demineralization. A similar trend was observed for the BET specific surface area. However, increasing the temperature further to 850 °C did not result in additional improvements in adsorption performance. In contrast, mass yield decreased steadily with increasing temperature, from 92.40% at 600 °C to 71.14% at 850 °C. At 800 °C, the mass yield remained at 77.51%, indicating a substantial yet acceptable material loss considering the achieved restoration of adsorption properties.

From an economic perspective, regeneration temperatures above 800 °C appear less advantageous because they are expected to increase energy consumption while simultaneously reducing the quantity of recoverable carbon without providing measurable gains in adsorption performance. Higher temperatures may also accelerate carbon burn-off, promote granule degradation, increase the generation of fine particles, and reduce the amount of material suitable for reuse. Therefore, based on the combined evaluation of adsorption parameters, mechanical strength, mass yield, and the I_SM_ index, regeneration at 800 °C followed by demineralization with a 1.75% HCl solution can be regarded as the most sustainable option among the investigated conditions. This treatment provided the highest recovery of adsorption properties while avoiding the unnecessary material losses observed at 850 °C.

In practical applications, a comprehensive economic assessment should additionally consider the cost of virgin activated carbon, thermal-regeneration operating costs, expenditures related to acid consumption and rinsing water, the quantity of regenerated material recovered, the number of achievable regeneration cycles, waste-management savings, and reductions in replacement costs. Inclusion of these factors in future studies would enable a more complete evaluation of the economic and environmental benefits associated with WG-12 regeneration under industrial conditions.

For full-scale implementation, several engineering aspects should also be addressed, including transportation of spent and regenerated carbon, treatment of acidic wastewater generated during demineralization, neutralization of rinsing effluents, potential changes in particle-size distribution, and compatibility of regenerated carbon with existing backwashing procedures. In particular, abrasion testing under simulated backwashing conditions and long-term cyclic regeneration studies are required before the proposed approach can be implemented in routine plant operation.

The findings of this study demonstrate the technical feasibility of a single regeneration cycle; however, they do not establish the long-term service life of the carbon during repeated regeneration and reuse. Future research should therefore focus on multi-cycle regeneration experiments, adsorption tests using representative target contaminants, and pilot-scale validation under hydraulic conditions representative of full-scale granular activated carbon systems.

### 3.9. Structural and Surface Morphological Changes in WG-12 Activated Carbon Following Operation and Regeneration

**Figure 10:**
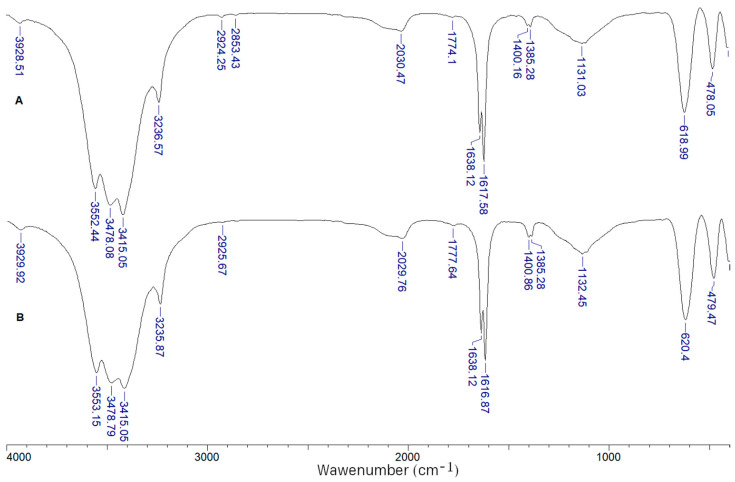
FTIR spectra of spent activated carbon used for contaminant removal (**A**) and regenerated activated carbon (**B**), recorded using KBr pellets in the 4000–400 cm^−1^ range.

The FTIR spectra obtained for the spent activated carbon, thermally regenerated samples, and samples subjected to regeneration followed by demineralization are presented in Figure 10. The spectra were analyzed within the wavenumber range of 4000–400 cm^−1^ to identify changes in the surface chemistry resulting from thermal treatment and acid washing.

A broad absorption band observed in the region of 3200–3600 cm^−1^ was assigned to the stretching vibrations of hydroxyl groups (–OH) associated with adsorbed water, phenolic groups, and carboxylic functionalities present on the carbon surface. The intensity of this band decreased after thermal regeneration, indicating partial removal of oxygen-containing surface groups and moisture during high-temperature treatment.

Bands located near 2920 cm^−1^ and 2850 cm^−1^ corresponded to asymmetric and symmetric stretching vibrations of aliphatic C–H bonds. These bands were more pronounced in the spent carbon, reflecting the presence of adsorbed organic contaminants accumulated during service. Their gradual reduction after regeneration confirms effective removal of residual organic compounds and decomposition products from the carbon surface.

The absorption band observed in the region of 1700–1720 cm^−1^ was attributed to C=O stretching vibrations originating from carboxylic acids, ketones, aldehydes, and lactones. Thermal regeneration reduced the intensity of this band, suggesting decomposition or transformation of oxygen-containing functional groups at elevated temperatures. Similar observations have been reported for thermally regenerated activated carbons, where high-temperature treatment promotes decarboxylation and deoxygenation reactions.

Signals appearing between 1580 and 1620 cm^−1^ were associated with C=C stretching vibrations within aromatic structures forming the carbon matrix. These bands remained relatively stable throughout the regeneration process, indicating preservation of the fundamental aromatic framework despite exposure to elevated temperatures.

In the region of 1000–1300 cm^−1^, several bands corresponding to C–O stretching vibrations in phenolic, alcoholic, ether, and ester groups were identified. The decrease in their intensity after regeneration further supports the removal or transformation of oxygen-containing functionalities. Following demineralization, these bands became more distinct, likely because acid treatment removed inorganic deposits that partially masked the surface functional groups.

The low-wavenumber region below 900 cm^−1^ contained bands associated with out-of-plane aromatic C–H vibrations and mineral components present in the activated carbon. Noticeable changes in this region after acid treatment indicate effective dissolution of inorganic compounds deposited during operation and regeneration.

Comparison of the spectra obtained after thermal regeneration at different temperatures showed that the most significant reduction in bands associated with adsorbed organic contaminants occurred between 600 °C and 800 °C. Increasing the temperature to 850 °C produced only minor additional spectral changes, suggesting that most removable surface deposits had already been eliminated at 800 °C. This observation is consistent with the adsorption-performance results, which also indicated that regeneration efficiency reached a maximum at approximately 800 °C.

The FTIR analysis therefore confirms that thermal regeneration effectively removed organic contaminants and altered selected oxygen-containing surface groups, while demineralization additionally eliminated inorganic deposits remaining within the pore structure. The combined treatment enhanced accessibility of the adsorption surface without causing substantial disruption of the aromatic carbon framework responsible for the structural stability of the activated carbon.

**Figure 11:**
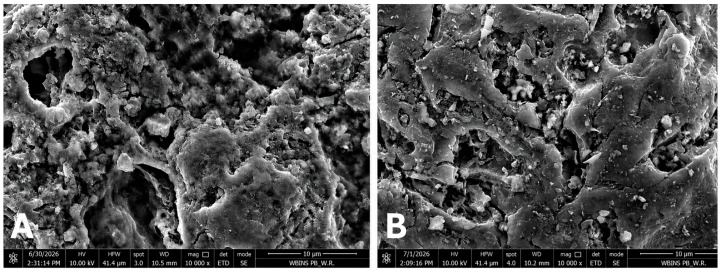
Surface morphology of WG-12 granular activated carbon observed by SEM: (**A**) sample after 5 years of operation in a water-treatment system; (**B**) sample after thermal regeneration followed by demineralization with a 1.75% HCl solution. Magnification: ×10,000.

SEM images (Figure 11) confirm the mechanism responsible for changes in the properties of WG-12 activated carbon during operation and regeneration. Long-term use resulted in coverage and partial blockage of the porous surface by accumulated deposits and adsorbed contaminants (Figure 11A). In contrast, thermal regeneration combined with HCl demineralization enabled partial removal of these deposits, reopening of the pore structure, and restoration of a more developed surface morphology (Figure 11B). These observations are consistent with the iodine number and BET specific surface area results, which demonstrated recovery of the adsorption potential of the regenerated activated carbon.

### 3.10. Limitations of the Study and Future Research Directions

Several limitations of the present study should be acknowledged when interpreting the results. First, although the eight samples collected from full-scale adsorption beds captured operational variability under practical conditions, they should not be regarded as a substitute for comprehensive laboratory repeatability testing involving multiple analytical replicates for each regeneration variant. Second, the evaluation of regeneration performance was based primarily on the iodine number (IN) and BET specific surface area (S_BET_), which are widely accepted indicators of adsorption capacity but do not directly reflect the removal efficiency of specific contaminants. Consequently, adsorption tests involving representative water pollutants, such as natural organic matter, disinfection by-product precursors, pesticides, pharmaceuticals, or other micropollutants, were beyond the scope of this study.

An additional limitation is that only a single regeneration cycle was investigated. Therefore, the long-term performance and service life of WG-12 activated carbon subjected to repeated regeneration remain unknown. Furthermore, the demineralization stage was evaluated using only one HCl concentration and one contact time. Optimization of acid concentration, treatment duration, rinsing requirements, and wastewater-management procedures may further improve the overall efficiency and sustainability of the process.

Future research should focus on evaluating the performance of WG-12 activated carbon over multiple regeneration cycles to determine the cumulative effects of thermal treatment and demineralization on adsorption properties, mechanical strength, and material losses. Additional studies should include adsorption experiments using representative target contaminants, abrasion tests under simulated backwashing conditions, and pilot-scale validation under hydraulic conditions representative of full-scale granular activated carbon (GAC) filtration systems.

To support industrial implementation, future investigations should also incorporate a comprehensive techno-economic and environmental assessment. Such analyses should consider energy consumption, acid and water requirements, wastewater treatment and neutralization, transportation costs, regeneration yield, the avoided purchase of virgin activated carbon, and the reduction in waste generation achieved through regeneration. Inclusion of these factors would provide a more complete assessment of the practical feasibility and sustainability of the proposed regeneration strategy.

## 4. Conclusions

The present study evaluated the regeneration of spent WG-12 activated carbon using thermal treatment at temperatures of 600, 700, 800, and 850 °C, followed by optional demineralization with a 1.75% HCl solution. The effectiveness of the regeneration process was assessed based on adsorption properties, physicochemical characteristics, mechanical strength, mass yield, and FTIR and SEM analyses.

1. The results demonstrated that regeneration temperature had a significant influence on the recovery of adsorption performance. Increasing the temperature from 600 °C to 800 °C resulted in systematic improvements in both the iodine number and BET specific surface area. The highest values were obtained at 800 °C, where the iodine number reached 858.1 mg/g after thermal regeneration and 903.9 mg/g after regeneration followed by demineralization. Under the same conditions, the BET specific surface area reached 806.9 m^2^/g and 883.6 m^2^/g, respectively.
2. Demineralization substantially enhanced regeneration effectiveness by removing inorganic deposits accumulated within the pore structure. For all investigated temperatures, acid-treated samples exhibited higher iodine numbers and larger specific surface areas than samples subjected solely to thermal regeneration. The greatest improvement was observed at 800 °C, confirming the beneficial effect of combining thermal regeneration with post-treatment demineralization.
3. Mechanical strength remained at a high level for all regenerated samples, although a gradual decrease was observed with increasing regeneration temperature. Despite this reduction, the values remained suitable for practical application, indicating that the structural integrity of the activated carbon was largely preserved throughout the regeneration process.
4. Mass-yield analysis revealed a progressive decrease in material recovery with increasing temperature, reflecting intensified carbon burn-off and removal of accumulated deposits. While higher temperatures improved adsorption performance, temperatures above 800 °C resulted in additional material losses without providing corresponding improvements in adsorption properties.
5. The strong positive correlation observed between the iodine number and BET specific surface area, together with the negative correlation between adsorption parameters and bulk density, confirms that regeneration restored accessibility of the pore system through removal of contaminants and mineral deposits. FTIR analysis further demonstrated the elimination of adsorbed organic compounds and changes in oxygen-containing surface groups while preserving the aromatic carbon framework.
6. Evaluation using the adsorption–mechanical trade-off index (I_SM_) identified regeneration at 800 °C followed by demineralization as the most effective treatment variant. Under these conditions, the regenerated carbon achieved the best balance between adsorption-property recovery and mechanical-strength retention, resulting in an I_SM_ value of 93.7%.
7. Overall, the findings indicate that thermal regeneration at approximately 800 °C, combined with subsequent demineralization, represents the optimal strategy for restoring the performance of spent WG-12 activated carbon. This approach enables substantial recovery of adsorption capacity while maintaining satisfactory mechanical properties and acceptable material yield, thereby supporting the sustainable reuse of activated carbon in industrial water-treatment applications.
8. The proposed I_SM_ index should be interpreted as an auxiliary adsorption-mechanical screening tool. It is useful for comparing the tested regeneration variants, but it should be combined with mass yield, cost indicators, cyclic durability and target-pollutant adsorption tests before final industrial recommendations are formulated.

## Figures and Tables

**Table 1:** Physicochemical parameters of WG-12 activated carbon.

No.	Parameter	Symbol	Unit	Value
1	Specific surface area	S_BET_	m^2^/g	min. 900
2	Iodine number	IN	mg/g	min. 950
3	Bulk density	ρ	kg/m^3^	min. 380
4	Mechanical strength	MS	%	min. 95
5	External appearance	-	-	black cylindrical granules

## Data Availability

The original contributions presented in this study are included in the article. Further inquiries can be directed to the corresponding author.
